# Depression links to unstable resting-state brain dynamics: insights from hidden markov models and functional network variability

**DOI:** 10.1017/S0033291725101001

**Published:** 2025-07-17

**Authors:** Li Geng, Qiuyang Feng, Xueyang Wang, Jiangzhou Sun, Shuang Tang, Hui Jia, Yu Li, Jiang Qiu

**Affiliations:** 1 Key Laboratory of Cognition and Personality (SWU), Ministry of Education, Chongqing, China; 2 Faculty of Psychology, Southwest University (SWU), Chongqing, China; 3 College of International Studies, Southwest University (SWU), Chongqing, China

**Keywords:** brain dynamics, depression, hidden markov model, resting-state fMRI, temporal variability

## Abstract

**Background:**

Depression is closely associated with abnormalities in brain function. Traditional static functional connectivity analyses offer limited insight into the temporal variability of brain activity. Recent advances in dynamic analyses enable a deeper understanding of how depression relates to temporal fluctuations in brain activity.

**Methods:**

This study utilized a large resting-state functional magnetic resonance imaging dataset (*N* = 696) to examine the association between brain dynamics and depression. Two complementary approaches were employed. Hidden Markov modeling (HMM) was used to identify discrete brain states and quantify their temporal switching patterns; temporal variability was computed within and between large-scale functional networks to capture time-varying fluctuations in functional connectivity.

**Results:**

Depression scores were positively associated with switching rate and negatively associated with maximum fractional occupancy. Furthermore, depression scores were significantly associated with greater temporal variability both within and between networks, with particularly strong effects observed in the default mode network, ventral attention network, and frontoparietal network. Together, these findings suggest that individuals with higher depression scores exhibit more unstable brain dynamics.

**Conclusion:**

Our findings reveal that individuals with higher depression levels exhibit greater instability in brain state transitions and increased temporal variability in functional connectivity across large-scale networks. This instability in brain dynamics may contribute to difficulties in emotion regulation and cognitive control. By capturing whole-brain temporal patterns, this study offers a novel perspective on the neural basis of depression.

## Introduction

Depression represents a significant global mental health burden, exerting profound impacts on societal well-being and public health. In recent years, the total number of depression cases has steadily increased, with a rising incidence particularly observed in high-income countries and specific regions (Ren et al., 2020; Xiang et al., 2024). Notably, younger populations are more vulnerable to depression (Thapar, Eyre, Patel, & Brent, 2022). Despite continuous advancements in medical and psychological interventions, a substantial proportion of individuals with depression remain undiagnosed and untreated in the early stages, exacerbating the overall disease burden. Consequently, the early identification and intervention of at-risk populations to reduce the incidence of depression have become critical challenges in depression prevention and management (Funkhouser et al., 2024; Petito et al., 2020).

Due to the complexity and high heterogeneity of depression, relying solely on behavioral assessments and clinical scales may be insufficient to comprehensively capture its pathological characteristics (Wu et al., 2023). Therefore, investigating its neural underpinnings not only facilitates a deeper understanding of the underlying mechanisms but also provides a neurobiological basis for early intervention. Existing research has demonstrated that depression is closely associated with abnormalities in brain function, particularly in key regions involved in emotion regulation, cognitive control, and self-referential processing, such as the prefrontal cortex, anterior cingulate cortex, and amygdala (Ebneabbasi et al., 2021; Hagen et al., 2025; Veer et al., 2010). However, most studies have primarily employed traditional static functional connectivity (sFC) analyses, which often overlook the dynamic changes and transient fluctuations in brain states during moment-to-moment transitions (Hutchison et al., 2013). In contrast, dynamic analysis captures the continuous temporal variations in brain activity, effectively overcoming the constraints of static analyses and providing novel insights into transient network reorganization and its relationship with clinical symptoms (Chang & Glover, 2010; Zhang et al., 2016). Brain dynamics have been increasingly recognized in recent years as a crucial aspect of understanding brain functional organization (França et al., 2024; Rolls, Cheng, & Feng, 2021).

Temporal variability in brain activity provides a more precise characterization of the interactions and coordination flexibility between different brain regions, capturing the spontaneous recurrence of functional connectivity patterns (Sun et al., 2019; Zhang et al., 2016). Given the moment-to-moment variability observed in depressive symptoms, such as shifts in affective state and attentional focus, dynamic measures are well-suited to capture the fluctuating nature of brain function in depression. As such, it serves as an effective tool for understanding the cognitive demands and emotional processing mechanisms in individuals with depression (Kaiser et al., 2016; Sun et al., 2024; Wu et al., 2022). Furthermore, the integration of machine learning with dynamic functional connectivity (dFC) analysis holds promise for enhancing early diagnosis and treatment assessment of depression – an advantage that sFC struggles to achieve (Dini et al., 2021; Wu et al., 2011). However, existing dFC studies have reported inconsistent findings regarding abnormalities in depression-related brain networks, with divergent results on whether dFC is increased or decreased in different regions, leaving the pathological mechanisms insufficiently understood (Sun et al., 2024). Therefore, further investigation into the dynamic characteristics of brain function in depression is of critical importance.

To deepen our understanding of these dynamic processes, it is necessary to further explore how depression relates to brain stability and variability. Moderate variability in brain functional dynamics plays a crucial role in maintaining a balance between information integration and flexible adaptation, thereby supporting cognitive function and emotion regulation (Cohen, 2018). Similarly, an optimal duration of state dwell time helps sustain metastability, allowing for more efficient and comprehensive information processing (Li, Lu, & Yan, 2020; Safron, Klimaj, & Hipólito, 2022). However, while greater brain flexibility is generally associated with improved cognitive function, excessive flexibility may also have negative consequences. When variability exceeds an optimal range, the coordination of neural networks may decline, leading to continuous redistribution of cognitive resources, which not only increases cognitive load but also contributes to emotional distress (Dinstein, Heeger, & Behrmann, 2015; Kucyi et al., 2017). For instance, Betzel et al. reported that positive emotions are associated with reduced flexibility in the dorsal attention network (DAN), suggesting that lower flexibility in certain contexts may facilitate emotional stability (Betzel, Satterthwaite, Gold, & Bassett, 2017). Additionally, excessively rapid state switching has been linked to attentional lapses, emotional instability, and increased cognitive load (Mora-Sánchez, Dreyfus, & Vialatte, 2019). Furthermore, excessive fluctuations within specific brain regions or between networks may undermine functional stability, making individuals more susceptible to emotional disturbances and cognitive impairments, ultimately increasing the risk of psychiatric disorders (Demirtaş et al., 2016; Dini et al., 2021; Gao et al., 2024; Long et al., 2020).

In summary, this study aims to investigate the dynamic characteristics of brain activity associated with depression. To this end, we employed two complementary approaches to characterize brain dynamics. First, we applied a hidden Markov model (HMM) to estimate the frequency of brain state transitions. Second, we used the conventional sliding-window method to quantify temporal variability in brain activity. While HMM provides detailed information on brain state switching, temporal variability further elucidates the dynamic fluctuations within and between brain networks. By integrating these two approaches, we can simultaneously capture transient state transitions and track the evolution of network structures over time, offering a multidimensional perspective on the neural dynamics underlying depression. We hypothesize that depression may be associated with instability in brain states, characterized by frequent state transitions, a lack of dominant states, and greater variability in functional connectivity (FC) within and between networks, particularly in key systems such as the default mode network (DMN).

## Methods

### Participants

The brain imaging and psychological questionnaire data used in this study were derived from our ongoing gene–brain behavior (GBB) project. For more details, refer to previous studies(Chen et al., 2019; Liu et al., 2024). All participants were recruited from Southwest University, Chongqing, China. They self-reported no history of mental illness or brain injury and received financial compensation upon completing all assessments. The study was approved by the Ethics Committee of the Brain Imaging Center at Southwest University. After excluding participants with mean head motion exceeding 0.2 mm, a total of 696 participants were included in the final analysis, comprising 204 males and 492 females, with a mean age of 19.42 ± 1.38 years.

### Measures

*Beck Depression Inventory-Second Edition*(Beck, Steer, & Brown, 1996). The Beck Depression Inventory-Second Edition (BDI-II) was used to measure participants’ depression scores in the current study. The BDI-II comprises 21 items, each rated on a 4-point Likert scale ranging from 0 (“none”) to 3 (“extremely severe”). Previous research has demonstrated good reliability for this scale in similar age groups, with a Cronbach’s alpha of 0.90 (Storch, Roberti, & Roth, 2004).

### Image acquisition and preprocessing

Brain imaging data were obtained using a Siemens 3 T Trio scanner (Siemens Medical System, Erlangen, Germany) at the Brain Imaging Center of Southwest University. Resting-state fMRI data were obtained using a gradient-echo echo-planar imaging (GRE-EPI) sequence with the following parameters: repetition time (TR) = 2,000 ms, echo time (TE) = 30 ms, flip angle (FA) = 90°, field of view (FOV) = 220 × 220 mm^2^, slices = 32, thickness = 3 mm, interslice gap = 1 mm, and voxel size = 3.4 × 3.4 × 4 mm^3^. Using a magnetisation-prepared rapid acquisition gradient-echo (MPRAGE) sequence, three-dimensional T1-weighted structural images with high resolution were acquired: TR = 1900 ms, TE = 2.52 ms, FA = 9°, segments = 176, FOV = 256 × 256 mm^2^, thickness = 1 mm, and voxel size = 1 × 1 × 1 mm^3^.

FMRIPrep (Esteban et al., 2019) based on Nipype (Gorgolewski et al., 2011) was used to preprocess the functional image data with the following parameters. Slice-timing correction was performed with AFNI’s 3dTshift(RRID: SCR_005927), followed by head motion correction using estimated motion parameters. The corrected BOLD time series were resampled to native space, and the BOLD reference image was co-registered to the T1-weighted structural image using boundary-based registration (FreeSurfer) and FLIRT with six degrees of freedom (Greve & Fischl, 2009). Physiological noise was addressed using CompCor (Behzadi, Restom, Liau, & Liu, 2007), extracting five principal components each from white matter and cerebrospinal fluid masks. Framewise displacement (FD) and DVARS were calculated as motion-related metrics. Nuisance regression was then performed to remove motion parameters, physiological noise components, and linear trends. Temporal filtering was applied with a bandpass range of 0.008–0.09 Hz to reduce low-frequency and high-frequency noise(Hallquist, Hwang, & Luna, 2013). After preprocessing, the BOLD data were normalized to the MNI152NLin2009cAsym template and spatially smoothed with a Gaussian kernel of 6-mm full-width at half-maximum.

### Hidden Markov model

The Schaefer 400 Parcels Atlas (17 networks) was employed to define cortical regions of interest, dividing the cortex into 17 distinct functional networks(Schaefer et al., 2018). The preprocessed time series data were standardized and concatenated into a matrix with dimensions of (696 participants × 242 volumes) × 17, where 17 corresponds to the mean signal extracted from the respective network masks. This resulting matrix was subsequently used as input for the Hidden Markov Model (HMM).

**Figure 1.**
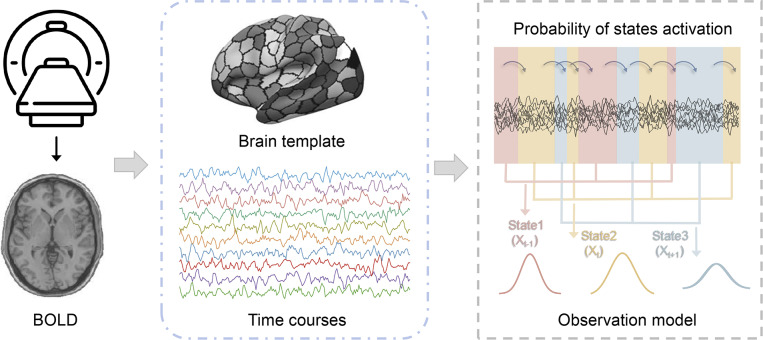
Workflow of fMRI data preprocessing and dynamic state analysis using HMM. MRI data were acquired and preprocessed to generate time-series signals. These signals were segmented into regional time series using a brain atlas template, and subsequently integrated into functional network time series. Functional network parcellation was based on the Schaefer 400 Parcel Atlas, which divides the entire brain into 17 specific functional networks. The resulting time-series data were subsequently analyzed using the HMM model. HMM assumes that the time series can be described by a finite number of hidden states, which are mutually exclusive in time and recur intermittently (as shown in the figure, with red, yellow, and blue representing three distinct states). The HMM output includes state activation probabilities at each time point and state-specific parameter estimates, revealing the dynamic properties of neural activity. Additionally, the probability of the hidden state at the current time point (*X_t_*) depends on the state at the previous time point (*X*_*t* − 1_), reflecting the temporal dependencies inherent to the model.

The present study used the HMM-MAR toolbox (Vidaurre et al., 2016) to infer an HMM from resting-state time series data (https://github.com/OHBA-analysis/HMM- MAR). HMM is an unsupervised machine learning method that segments observed time series into discrete hidden functional states, which are mutually exclusive in time and recur intermittently. These states are mutually exclusive in time and recur intermittently. Model parameters were estimated using a variational Bayes approach and optimized by minimizing free energy to ensure robust model fitting(Zhang et al., 2024). The HMM assigns state probabilities to each time point in the time series and estimates the parameters of the states, as illustrated in Figure 1.

Previous studies have suggested that selecting 8–12 states for modeling fMRI dynamics achieves a balance between model complexity and reproducibility(Vidaurre et al., 2018; Vidaurre, Smith, & Woolrich, 2017). Based on this, we conducted 10 independent runs of the HMM for model orders ranging from 8 to 12 states to account for variability between iterations. As shown in Supplementary Figure S1, the eight-state solution demonstrated the highest stability across all iterations. Therefore, we selected an eight-state configuration for the final analysis. To ensure robust estimation, we performed 10 iterations of the model and selected the iteration with the lowest free energy as the optimal solution. The HMM-MAR toolbox generates a range of outputs to estimate the characteristics of different HMM states. This study focused on two key metrics. The first is fractional occupancy (FO), which quantifies the proportion of total time spent in a specific state. The second is switching rate (SR), representing the frequency of state transitions within an individual’s time series and serving as an indicator of network stability (Toffoli et al., 2024).

### Network temporal variability

The temporal variability reflects the dynamic reorganization of brain activity over time, as captured by changes in FC across successive time windows(Zhang et al., 2016). Within-network variability quantifies the extent to which the FCs within a specific brain network change across time, while between-network variability measures the consistency of FC patterns between two distinct brain networks over time. Higher variability indicates greater differences in FCs across time windows, reflecting increased flexibility or instability of the network. In this study, the Schaefer400 parcellation template was employed to calculate both the within-network and between-network variability for 17 predefined networks. For a specific network *m*, the BOLD time series of all *k* ROIs within the network were extracted and divided into *n* non-overlapping time windows of length *l.* Within each time window *i*, the FCs within the network were calculated as *F_m,i_.* The within-network variability of network *m* is defined as:(see Figure 4a)

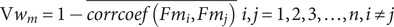



Similarly, for two networks *l* and *p*, the FCs between these two networks in each time window *i* were represented as *Fm_i,l_m_i,p_.* The between-network variability is defined as:(see Figure 4c)

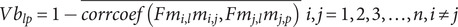



To avoid the influence of specific window size selection on the results, multiple window lengths were tested, ranging from *l* = 20, 22, 24, …, 40, and the mean variability across all tested window lengths was computed as the final value(Sun et al., 2019).

## Results

### Association between depression scores and dynamic metrics from HMM models

**Figure 2.**
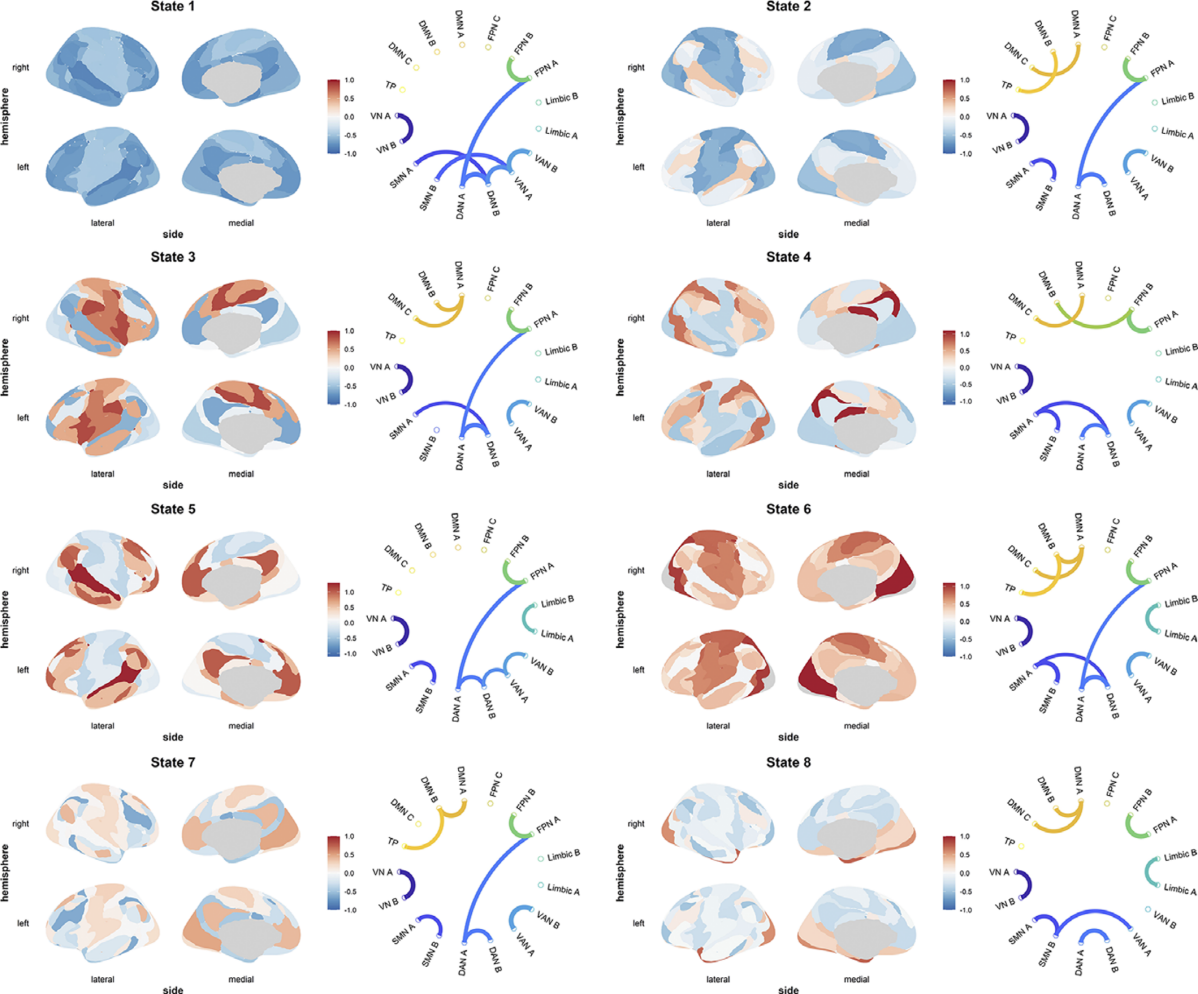
Spatial and functional connectivity profiles of brain states identified by the hidden Markov model during the scan. For each brain state, the left panel displays the spatial distribution of average activation, representing the relative loading with respect to the mean activation. Blue indicates negative activation, while red indicates positive activation. The right panel illustrates the top 5% of positive functional connectivity, highlighting the strongest connections associated with each state.

We successfully identified 8 HMM states, with the fMRI signal distributions for each state shown in Figure 2 and Supplementary Figure S3. Some states, such as State 5 and State 6, exhibit significant positive activation in specific regions, including the prefrontal and frontotemporal areas, which may be associated with the high integration of local functions such as executive control, language processing, or emotion regulation. Other states, such as State 3, display complex cross-regional connectivity patterns, including significant connections between the DMN and the DAN, potentially reflecting dynamic coordination between large-scale brain networks involved in attention allocation or task switching.

The dynamic metrics of HMM states are derived from the temporal evolution of state probabilities inferred by the model. Supplementary Figure S2 illustrates the temporal dynamics of these states. SR quantifies the frequency of transitions between states over time and is calculated by dividing the total number of state switches by the total number of time points. A higher SR indicates more frequent state transitions, reflecting greater dynamic flexibility and possibly a less stable system. FO measures the proportion of time each hidden state is expressed during the entire time series. A higher FO indicates that the state is more stable or frequently expressed. Maximum Fractional Occupancy (MaxFO), an extension of FO, represents the highest FO among all states, providing a global perspective on the dominance of a single state over the entire time series. A lower MaxFO reflects a more dynamic and distributed system, where no single state dominates, whereas a higher MaxFO indicates a system predominantly remaining in one state with reduced flexibility. These metrics collectively describe the balance between dynamic flexibility and stability in brain activity, offering a quantitative framework to study individual differences and clinical conditions.

**Figure 3.**
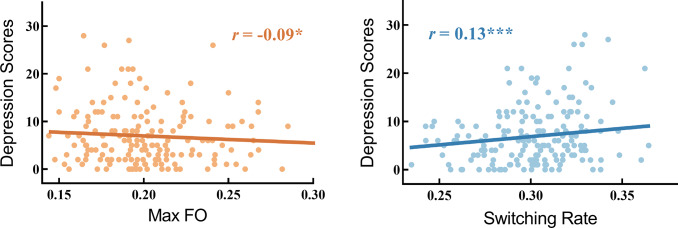
Relationships between MaxFO, Switching Rate, and depression scores. Significance levels are indicated as *p < 0.05 and ***p < 0.001. For clarity, only 1/4 of the data points were plotted in scatterplots, with every fourth point shown.

We calculated the correlation between the FO of each state and depression scores, controlling for sex, age, and mean head motion. The results showed that the FO of State 1 was positively associated with depression scores (*r* = 0.12, *p_fdr_* = 0.010). In contrast, the FO of States 4, 5, and 8 was negatively associated with depression scores, with *r* values ranging from −0.09 to −0.11 and *p_fdr_* values between 0.015 and 0.030. Supplementary Figure S4 illustrates the FO of each state, and detailed statistical results are provided in Supplementary Table S1. Furthermore, we found that MaxFO was negatively associated with depression scores (*r* = −0.09, *p* = 0.023). In contrast, SR was positively associated with depression scores (*r* = 0.13, *p* < 0.001). These results are visualized in Figure 3.

### Association between depression scores and network temporal variability

**Figure 4.**
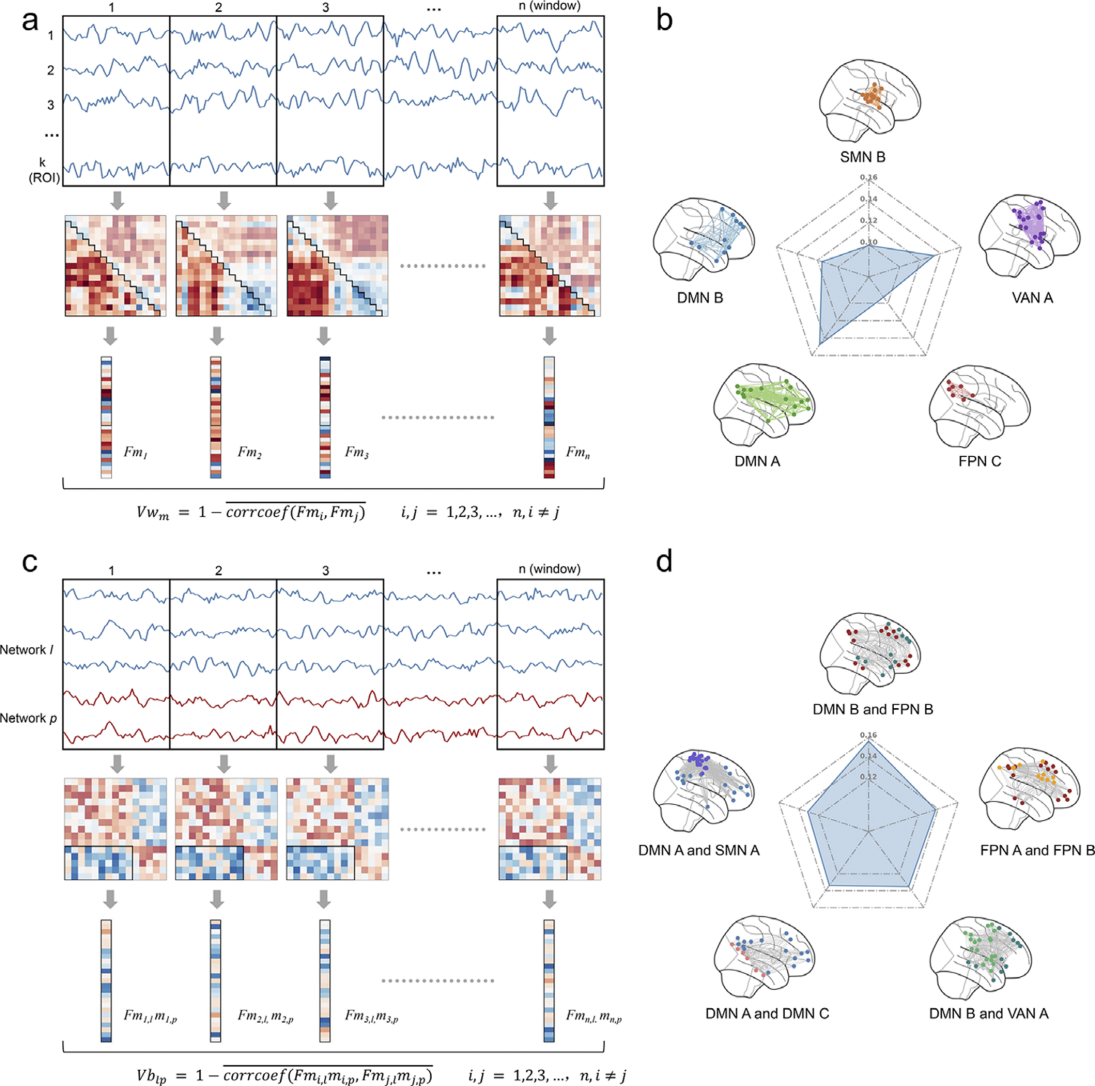
Relationships between within-network and between-network temporal variability and depression scores. (a) Calculation of within-network variability. The BOLD signals of ROIs within each network were divided into *n* nonoverlapping time windows of length *l.* Functional connectivity (FC) was calculated for each time window, and variability was estimated across all time windows. (b) Networks with within-network variability significantly associated with depression scores. (c) Calculation of between-network variability. Using a similar approach, the FC variability for each network pair was calculated, reflecting the dynamic changes in FC patterns between networks. (d) The five network pairs with the strongest correlations between between-network variability and depression scores. DMN, ‘Default Mode Network’; SMN, ‘Somatomotor Network’; VAN, ‘Ventral Attention Network’; FPN, ‘Frontoparietal Control Network’.

Based on the Schaefer 400 parcellation template, we calculated the within-network variability for 17 predefined networks and the between-network variability for 136 network pairs (17 × 16/2). Regarding the relationship between within-network variability and depression scores, after controlling for sex, age, and mean head motion, and applying FDR correction to the p-values, we found that the variability of the default mode network A (DMN A), default mode network B (DMN B), ventral attention network A (VAN A), Frontoparietal Control Network C (FPN C), and Somatomotor Network B (SMN B) were significantly positively associated with depression scores, with r-values ranging from 0.09 to 0.15 (Figure 4b). For the between-network variability, 46 network pairs showed significant positive correlations with depression scores (*r*-values ranging from 0.09 to 0.16). Figure 4d highlights the five network pairs with the strongest correlations, with r-values between 0.13 and 0.16. Detailed results are provided in Supplementary Tables S2 and S3.

**Figure 5.**
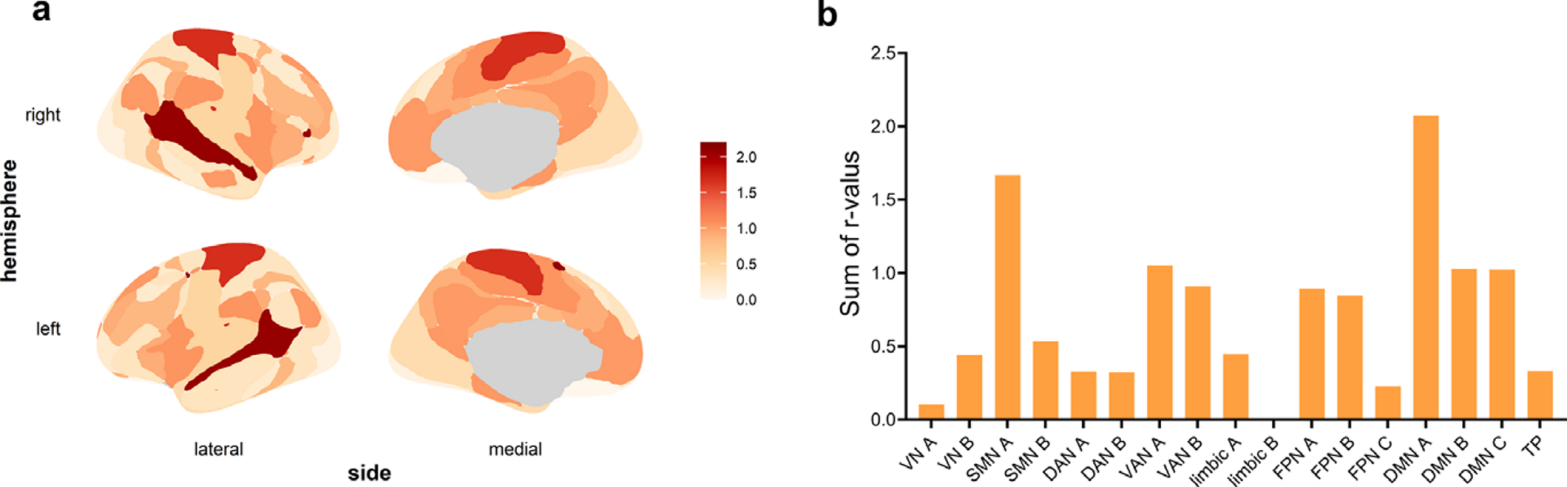
Contribution of individual networks to between-network variability associated with depression scores. (a) Spatial distribution of network-level contributions, displayed as cumulative *r*-values mapped onto the cortical surface. Warmer colors indicate higher contributions. (b) Bar plot showing the sum of *r*-values for each network, representing the cumulative correlation between each network’s between-network variability and depression scores. DMN, ‘Default Mode Network’; SMN, ‘Somatomotor Network’; VAN, ‘Ventral Attention Network’; FPN, ‘Frontoparietal Control Network’; TP, ‘Temporoparietal Network’.

To evaluate the contribution of individual networks to the observed between-network variability associated with depression scores, we aggregated the correlation coefficients (*r*-values) from significant network pairs to their corresponding networks. By summing the weighted contributions, we calculated cumulative *r*-values for each network, providing insights into which networks’ FC dynamics were more strongly associated with depression scores. The results revealed that the DMN contributed the most to these associations. The top five networks with the highest weights were DMN A, SMN A, VAN A, DMN B, and DMN C (Figure 5).

## Discussion

In this study, we employed two complementary approaches to characterize brain dynamics associated with depression. First, we used a Hidden Markov Model to capture latent patterns about brain state transitions, and then we calculated the temporal variability of functional connectivities within and between brain networks, examining their relationship with depression scores. The results indicate that individuals with higher depression scores tend to exhibit a higher SR and a lower maxFO, suggesting increased instability in brain state dynamics – characterized by a lack of dominant states and frequent transitions between states. Additionally, depression scores were significantly positively correlated with both within-network variability and between-network variability, with particularly strong associations observed in the DMN, frontoparietal network (FPN), and VAN. These findings deepen our understanding of the dynamic neural characteristics of depression and provide novel neuroimaging evidence that may inform future efforts in early diagnosis and intervention.

To further explore the dynamic neural characteristics associated with depression, we applied the HMM to resting-state fMRI data. In recent years, HMM has gained popularity due to its ability to accurately capture brain state transitions without the need for predefined time windows(Toffoli et al., 2024; Vidaurre et al., 2017). This study focuses on two key metrics: FO, which represents the proportion of time spent in a specific state and SR, which reflects the frequency of state transitions. In this study, we successfully identified eight HMM states. The results showed that FO of State 1 was positively correlated with depression scores, while FO of States 4, 5, and 8 was negatively correlated with depression scores. Additionally, the MaxFO was negatively correlated with depression scores, whereas the SR was positively correlated with depression scores.

Further analysis of the characteristics of each state revealed that State 1 is characterized by negative activation across the whole brain, particularly involving regions such as the prefrontal cortex and parietal lobe. The dwell time in this state was positively correlated with depression, suggesting that individuals with higher levels of depression may be in a “low-functioning” or “low-arousal” state. Consistent with a meta-analysis, depressed patients, compared to healthy individuals, exhibit lower functional activity in regions such as the prefrontal cortex, cingulate gyrus, and insula, potentially leading to deficits in cognitive control, emotion regulation, and self-referential processing, which in turn contributes to low mood and impaired attention (Fitzgerald, Laird, Maller, & Daskalakis, 2008). In contrast, State 4, State 5, and State 8 exhibit patterns of both positive and negative activation, with positively activated regions including the prefrontal cortex, supplementary motor area, and anterior cingulate cortex. These states involve FC within key networks such as the VAN, FPN, and DAN, as well as connectivity between the DMN, sensorimotor network (SMN), and visual network (VN). Thus, these states may reflect higher levels of cognitive and emotional regulation functions, such as focused attention, emotion regulation, and behavioral planning(Dixon et al., 2018; Petrican, Saverino, Rosenbaum, & Grady, 2015; Stevens, Hurley, & Taber, 2011). Accordingly, individuals with higher depression scores showed shorter dwell times in these states, suggesting potential impairments in executive function or deficits in emotional regulation.

Furthermore, depression scores were negatively correlated with MaxFO, indicating that individuals with higher levels of depression tend to have shorter durations of their dominant brain states. Additionally, depression scores were positively correlated with the SR, suggesting that these individuals experience more frequent transitions between different states, lacking a stable dominant state, and exhibiting a pattern of unstable and rapidly shifting brain activity. Previous studies have shown that dynamic brain activity is closely related to cognitive processes such as attention and inhibitory control (Cohen, 2018; Fong et al., 2019). Therefore, the stability of brain physiological signals may reflect underlying pathological mechanisms in individuals with mental disorders (Ingabire et al., 2022). For example, prior research has found that the stability of DMN connectivity is significantly reduced in patients with major depression (Wise et al., 2017). Other studies have also revealed associations between unstable brain states and disorders such as bipolar disorder and schizophrenia (Perry, Roberts, Mitchell, & Breakspear, 2019; Zhong et al., 2024). Thus, the findings of this study may reflect dynamic instability in cognitive and emotional regulation processes among individuals with depression, suggesting difficulties in maintaining continuous functional integration and effective emotional regulation.

Additionally, we examined the relationship between temporal variability in brain functional network connectivity and depression. The results showed that depression scores were most strongly positively correlated with temporal variability within the DMN, VAN, and FPN. Similarly, between-network variability analysis yielded comparable results, with weight analysis indicating that DMN, SMN, and VAN contributed most significantly.

The DMN is typically associated with attention, self-referential thinking, and introspection, and its excessive variability may reflect instability in attention and cognitive control (Kucyi et al., 2017). Consistent with prior findings, increased DMN temporal variability has been linked to frequent mind-wandering and impaired decision-making (Kucyi, Esterman, Riley, & Valera, 2016; Mowinckel et al., 2017). The VAN plays a crucial role in emotional awareness and selective attention to external stimuli, and excessive fluctuations in this network may lead to emotional dysregulation and attentional instability (Viviani, 2013). Additionally, previous studies have found that adolescents with higher depression scores exhibit increased intra-network connectivity in the VAN, potentially reflecting underlying neural mechanisms of stimulus-driven attentional abnormalities that contribute to a persistent focus on negative information (Liu et al., 2019). The FPN is primarily responsible for cognitive control and executive function, and excessive variability in its internal connectivity may reduce information integration efficiency, thereby impairing emotion regulation (Zanto & Gazzaley, 2013). Research has shown that dynamic variability between the FPN and DMN at rest is closely related to poorer cognitive flexibility (Douw et al., 2016), while another study found that patients with first-episode psychosis exhibit instability in dFC, which is considered a potential mechanism underlying cognitive control deficits (Briend et al., 2020). Overall, these findings suggest that heightened variability across key networks may collectively reflect functional dysregulation in emotional and cognitive processing among individuals with depression, providing new insights into the neuropathological mechanisms of the disorder.

However, this study has several limitations. First, although we investigated brain dynamics using resting-state data, the information obtained from resting-state fMRI remains limited. Future studies should incorporate task-based data to provide a more comprehensive understanding of dynamic brain changes in individuals with depression under different cognitive tasks. Second, our analysis was primarily based on variations in depression scores within a healthy population. Future research should validate and extend these findings in clinically diagnosed depression patients and other psychiatric populations. Lastly, this study focused solely on the dynamic neural characteristics associated with depression without developing a predictive or diagnostic model. Future studies should integrate multimodal and interdisciplinary approaches, such as machine learning, genetics, and physiological signal analysis, to develop more robust depression prediction models based on dynamic brain features.

## Conclusion

This study systematically explored the dynamic brain characteristics associated with depression. The results revealed that individuals with higher depression scores exhibited more frequent state switching and shorter maintenance of dominant states, reflecting greater instability in brain dynamics. Additionally, depression scores were significantly positively correlated with the temporal variability of both within-network and between-network FC, with the strongest associations observed in the DMN, FPN, and VAN. These findings improve our understanding of depression-related brain dynamics, provide new insights into the neural mechanisms underlying depression, and may offer potential neurobiological markers for early diagnosis and intervention.

## Supporting information

Geng et al. supplementary material Geng et al. supplementary material

## Supplementary material

The supplementary material for this article can be found at http://doi.org/10.1017/S0033291725101001.
