# Supplementary material for: Depression links to unstable resting-state brain dynamics: insights from hidden markov models and functional network variability
Source: Psychol Med. 2025 Jul 17;55:e200. doi: 10.1017/S0033291725101001 (PMC12315643; doi:10.1017/S0033291725101001)

Supplementary Information

**Depression Links to Unstable Resting-State Brain Dynamics: Insights from Hidden Markov Models and Functional Network Variability**

Geng et al.

**TableS1 Fractional Occupancy of HMM States and Correlation with Depression Scores**

| **State** | **Fractional occupancy(mean ± sd)** | ***r*** | ***p*** | ***p_fdr_*** |
| --- | --- | --- | --- | --- |
| State1 | 0.105 ± 0.068 | 0.122 | 0.001 | 0.010 |
| State2 | 0.122 ± 0.050 | 0.070 | 0.068 | 0.108 |
| State3 | 0.126 ± 0.043 | 0.046 | 0.230 | 0.307 |
| State4 | 0.126 ± 0.042 | -0.105 | 0.006 | 0.015 |
| State5 | 0.140 ± 0.039 | -0.105 | 0.006 | 0.015 |
| State6 | 0.103 ± 0.040 | -0.013 | 0.738 | 0.738 |
| State7 | 0.141 ± 0.042 | -0.018 | 0.632 | 0.722 |
| State8 | 0.138± 0.040 | -0.093 | 0.015 | 0.030 |

**TableS2 Networks with within-network variability significantly associated with depression scores.**

| **Network** | ***r*** | ***p*** | ***p_fdr_*** |
| --- | --- | --- | --- |
| somatomotorBAuditory | 0.099 | 0.009 | 0.038 |
| salienceVentralAttentionA | 0.134 | 4.17e-04 | 0.004 |
| frontoparietalControlC | 0.093 | 0.014 | 0.048 |
| defaultA | 0.148 | 9.70e-05 | 0.002 |
| defaultB | 0.116 | 0.002 | 0.013 |

**TableS3 Networks with between-network variability significantly associated with depression scores.**

| Network 1 | Network2 | ***r*** | ***p*** | ***p_fdr_*** |
| --- | --- | --- | --- | --- |
| frontoparietalControlB | defaultB | 0.156 | 3.56e-05 | 0.018 |
| frontoparietalControlA | frontoparietalControlB | 0.137 | 2.85e-04 | 0.018 |
| salienceVentralAttentionA | defaultB | 0.135 | 3.56e-04 | 0.018 |
| defaultA | defaultC | 0.134 | 4.17e-04 | 0.018 |
| somatomotorA | defaultA | 0.131 | 0.001 | 0.018 |
| salienceVentralAttentionB | defaultA | 0.129 | 0.001 | 0.019 |
| visualPeripheral | defaultA | 0.129 | 0.001 | 0.018 |
| salienceVentralAttentionA | defaultC | 0.126 | 0.001 | 0.018 |
| somatomotorA | defaultC | 0.126 | 0.001 | 0.018 |
| frontoparietalControlB | temporoparietal | 0.124 | 0.001 | 0.025 |
| salienceVentralAttentionA | salienceVentralAttentionB | 0.124 | 0.001 | 0.025 |
| salienceVentralAttentionA | defaultA | 0.124 | 0.001 | 0.020 |
| somatomotorBAuditory | salienceVentralAttentionA | 0.121 | 0.001 | 0.025 |
| salienceVentralAttentionB | defaultC | 0.118 | 0.002 | 0.020 |
| frontoparietalControlC | defaultA | 0.118 | 0.002 | 0.028 |
| salienceVentralAttentionA | limbicOrbitofrontal | 0.115 | 0.003 | 0.025 |
| defaultA | defaultB | 0.114 | 0.003 | 0.025 |
| dorsalAttentionA | defaultA | 0.113 | 0.003 | 0.025 |
| frontoparietalControlB | defaultC | 0.113 | 0.003 | 0.025 |
| salienceVentralAttentionB | defaultB | 0.113 | 0.003 | 0.025 |
| somatomotorA | frontoparietalControlA | 0.113 | 0.003 | 0.025 |
| somatomotorA | limbicOrbitofrontal | 0.112 | 0.003 | 0.025 |
| somatomotorBAuditory | frontoparietalControlA | 0.111 | 0.003 | 0.025 |
| visualPeripheral | defaultB | 0.111 | 0.003 | 0.025 |
| limbicOrbitofrontal | defaultC | 0.111 | 0.004 | 0.025 |
| dorsalAttentionB | salienceVentralAttentionB | 0.110 | 0.004 | 0.029 |
| dorsalAttentionB | frontoparietalControlA | 0.110 | 0.004 | 0.029 |
| frontoparietalControlB | defaultA | 0.109 | 0.004 | 0.031 |
| somatomotorBAuditory | limbicOrbitofrontal | 0.109 | 0.004 | 0.025 |
| dorsalAttentionA | frontoparietalControlA | 0.109 | 0.004 | 0.033 |
| salienceVentralAttentionA | frontoparietalControlB | 0.108 | 0.004 | 0.030 |
| somatomotorA | defaultB | 0.108 | 0.004 | 0.029 |
| salienceVentralAttentionB | frontoparietalControlC | 0.108 | 0.005 | 0.033 |
| somatomotorA | dorsalAttentionA | 0.105 | 0.006 | 0.037 |
| visualPeripheral | temporoparietal | 0.105 | 0.006 | 0.036 |
| salienceVentralAttentionB | frontoparietalControlA | 0.105 | 0.006 | 0.049 |
| salienceVentralAttentionA | frontoparietalControlA | 0.104 | 0.006 | 0.038 |
| frontoparietalControlA | defaultB | 0.103 | 0.007 | 0.036 |
| somatomotorA | salienceVentralAttentionB | 0.102 | 0.007 | 0.033 |
| dorsalAttentionB | defaultA | 0.102 | 0.007 | 0.033 |
| visualCentral | defaultC | 0.101 | 0.008 | 0.039 |
| defaultC | temporoparietal | 0.101 | 0.008 | 0.046 |
| somatomotorBAuditory | frontoparietalControlB | 0.097 | 0.011 | 0.035 |
| somatomotorBAuditory | defaultB | 0.095 | 0.013 | 0.037 |
| visualPeripheral | salienceVentralAttentionA | 0.094 | 0.013 | 0.048 |
| defaultB | defaultC | 0.093 | 0.014 | 0.046 |

**Figure S1 Mean similarity of gamma time courses across different numbers of states (*k*).** This figure illustrates the mean similarity of gamma time courses across different state configurations, ranging from 8 to 12 states. The y-axis represents the mean similarity, while the x-axis corresponds to the number of states (*k*). Among the tested configurations, the 8-state solution exhibited the highest mean similarity, supporting its selection as the optimal state number for further analysis.


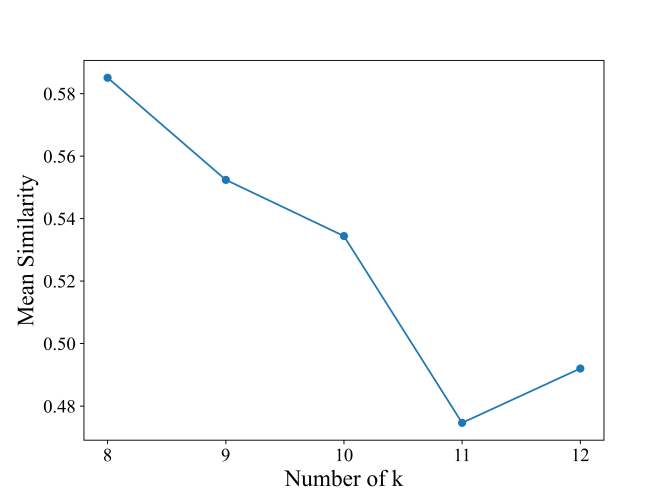


**Figure S2 Temporal dynamics of HMM-derived brain states during the scan**.The top panel shows the gamma probabilities for each of the 8 hidden states across all time points, where each color represents a specific brain state. The height of the gamma probability at a given time point reflects the likelihood of the corresponding state being active. The bottom panel illustrates the most expressed state at each time point, determined by the highest gamma probability. This visualization highlights the temporal transitions between different brain states and their dynamic reorganization over time.

**
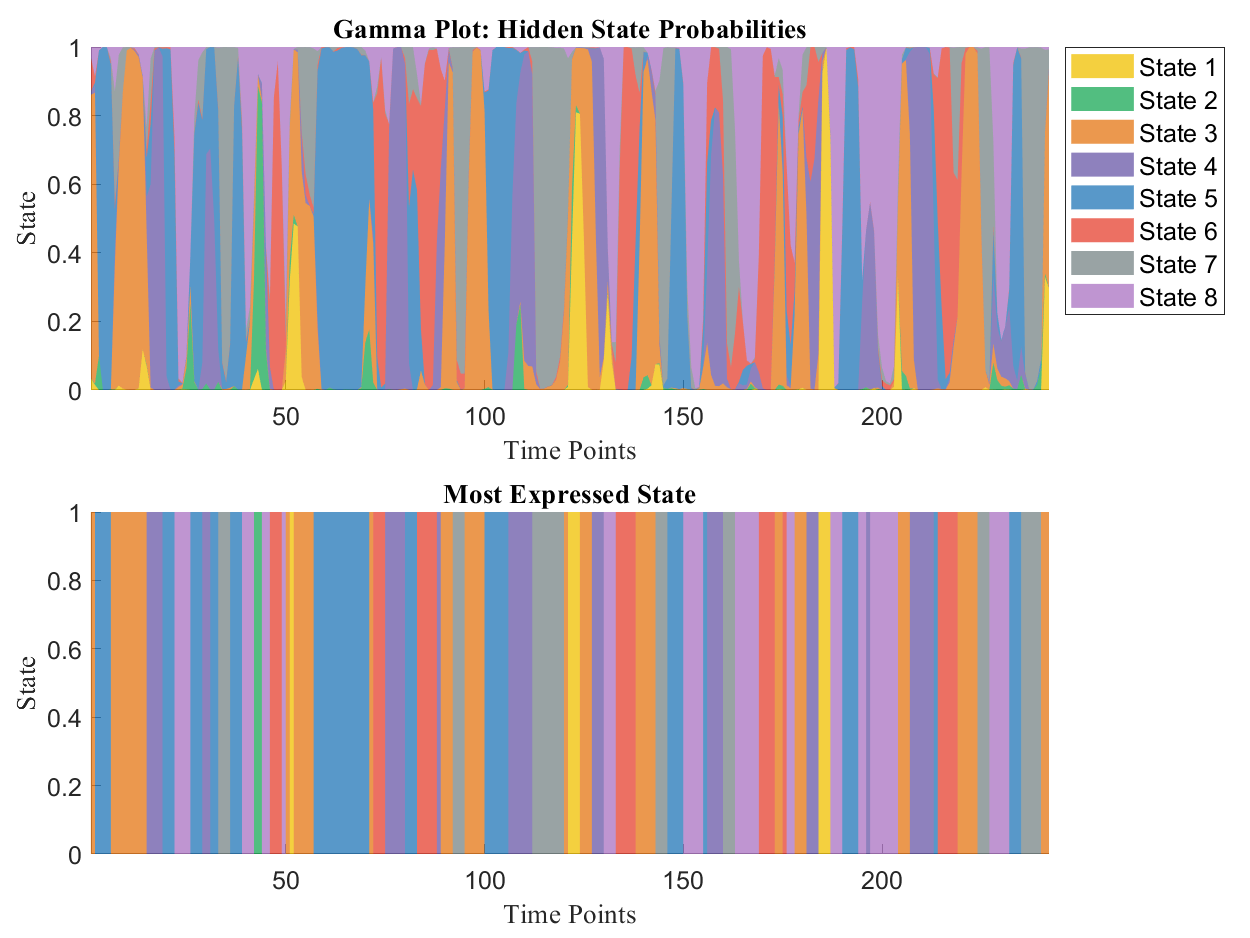
**

**Figure S3 The mean activation of HMM states.**


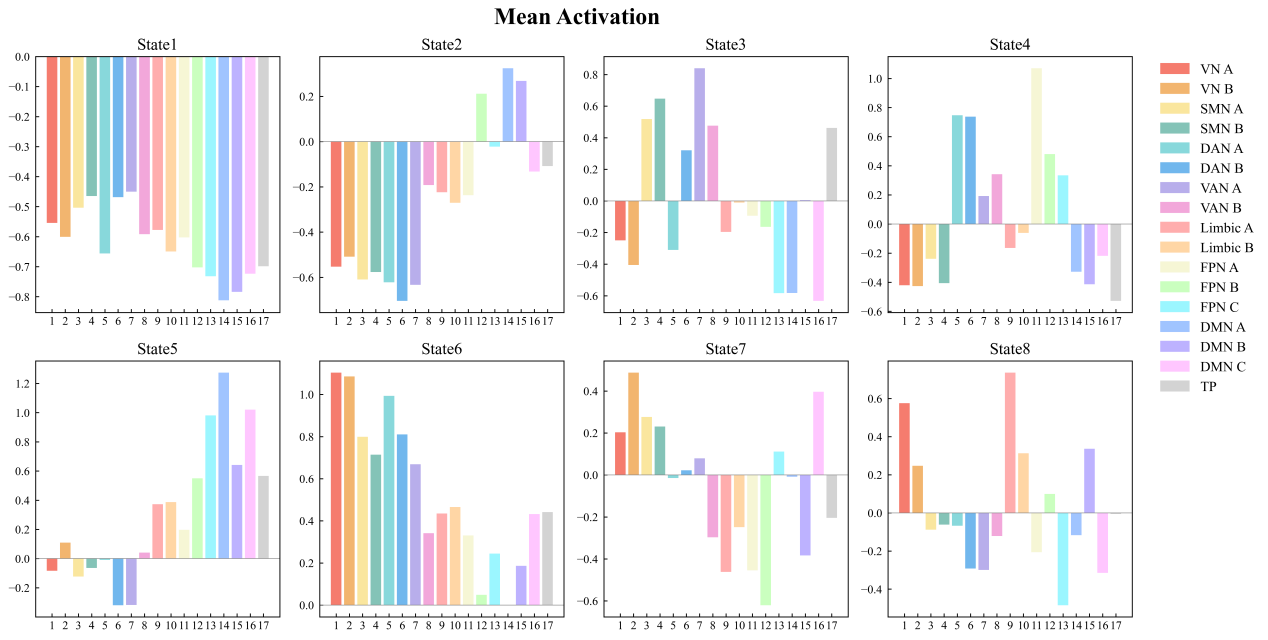


**Figure S4. Fractional Occupancy (FO) for each HMM state.** The violin plots display the distribution of FO across participants for each of the eight HMM states, overlaid with boxplots indicating the median and interquartile range (IQR). Individual data points represent the FO of each participant.


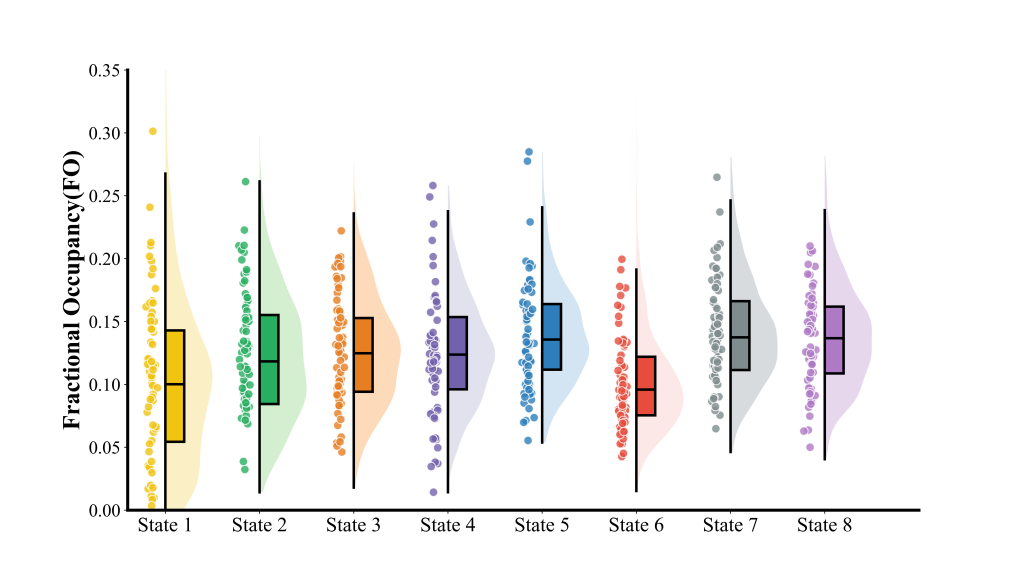

Supplement: Geng et al. supplementary material [file S0033291725101001sup001.docx]
